# Spontaneous Pneumomediastinum in a Healthy Pediatric Patient

**DOI:** 10.7759/cureus.17847

**Published:** 2021-09-09

**Authors:** Hebah Hassan, Lincoln Ferguson

**Affiliations:** 1 Internal Medicine, New York Institute of Technology College, Old Westbury, USA; 2 Pediatrics, Mount Sinai South Nassau Hospital, Oceanside, USA

**Keywords:** spontaneous pneumomediastinum, trauma, pediatrics, subcutaneous emphysema, mediastinitis, pneumomediastinum

## Abstract

Spontaneous pneumomediastinum (SPM) is a rare condition, especially in children with no predisposing factors. In the vast majority of patients, this condition is benign and self-limiting; however, there is always the possibility that serious and potentially life-threatening complications such as mediastinitis or cardiac tamponade could arise. Early recognition, prompt diagnosis, and appropriate management allow for ideal care and prevent unnecessary and excessive investigations in these patients. An eight-year-old female was admitted to the emergency department with SPM after swimming and no known predisposing lung conditions. The probable causative event was likely to be pressure changes in the alveoli during swimming. This is notable because the patient’s SPM occurred in the absence of an underlying cause such as asthma. The patient was admitted overnight for monitoring and pain control. The symptoms resolved the following day, along with a decrease in the size of the SPM on the chest X-ray. Physicians should be aware of the signs of SPM in young patients who present with chest pain in the absence of trauma or pulmonic disease. A review of literature highlighted the pathophysiology and recommended treatment course for similar cases.

## Introduction

A pneumomediastinum is a potentially life-threatening condition in which there is an abnormal presence of air in the mediastinum, between the lungs. The typical patient presents with a nonspecific, pleuritic chest pain accompanied by dyspnea [1]. In most cases, it is a benign, self-limiting disease that infrequently occurs in the absence of trauma - its incidence ranging from 1 in 800 to 1 in 42,000 pediatric cases with spontaneous pneumomediastinum (SPM) [2]. It is rarer to encounter primary SPM in pediatric patients. This sub-classification of SPM occurs when the patient has no pre-existing lung condition like asthma [3].

Since pneumomediastinums rarely occur, most of the literature describing this disease is found in individual case reports. Although the course of this disease is typically benign, hospitalization and observation are common due to possible significant or life-threatening complications [4]. This paper discusses the clinical characteristics of SPM and recommends appropriate management of SPM in pediatrics.

## Case presentation

An eight-year-old Caucasian female presented to the emergency department with chest pain and throat pain, which had persisted for 20 minutes and made her awaken from sleep crying. Her mother reported no medical history of these symptoms. The patient's symptoms started as she was swimming and diving in a public pool. She denied choking on water, swallowing pool water, or trauma. The patient appeared well and only complained of losing her voice. The patient’s mother stated that the patient was not given any medication prior to arrival and that the patient is a strong swimmer. Patient notes that her chest only hurts when she moves into a certain position, but nothing has made the pain better.

The patient has no significant medical history or family history, is up to date on vaccinations, and was not prescribed any regular medications. Her only known allergy is to cefadroxil. On presentation, the patient was afebrile with stable vitals, though she spoke in a hoarse voice and had slight tonsillar swelling. Her trachea was found to be tender and midline. In addition, the patient reported mild pain on hyperextension of her neck, and palpable mild subcutaneous emphysema was noted in the neck.

Treatment and management

**Figure 1:**
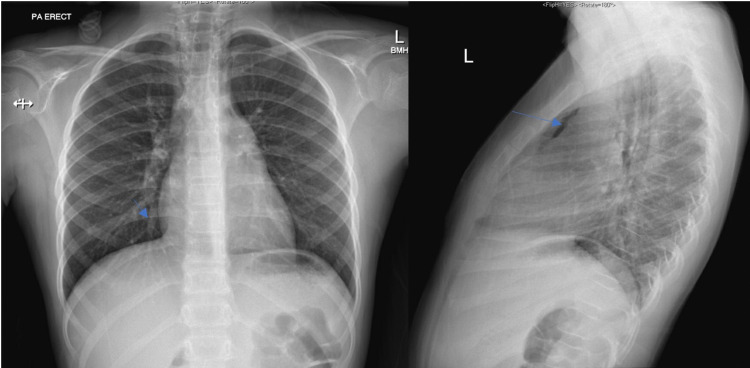
Initial Anterior/Posterior and Lateral Chest X-rays Air tracking into the soft tissues of the neck and continuous air in the superior mediastinum is seen in the anterior/posterior (left) and lateral (right) chest X-rays. No significant pericardial or pleural effusions are noted.

During her ER course, the rapid strep test was negative. A chest X-ray and chest CT was performed. The chest X-ray (PA + lateral) demonstrated a superior mediastinum pneumomediastinum (Figure 1). The heart was of normal size, and no significant pericardial or pleural effusions were seen. No blebs were noted on the chest CT. Furthermore, the patient’s O_2_ saturation remained 97-100%.

**Figure 2:**
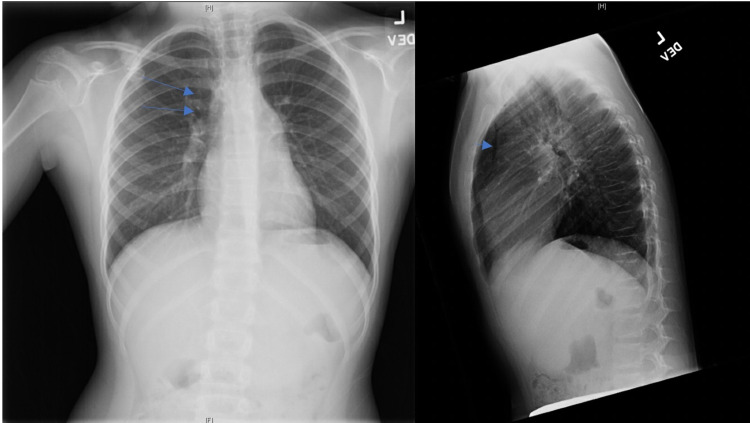
Follow-Up Anterior/Posterior and Lateral Chest X-ray There is a mild decrease in the size of the SPM noted in the anterior/posterior (left) and lateral (right) chest X-rays in comparison to that seen in Figure 1.

Acetaminophen suspension was administered; the patient’s pain was well controlled with acetaminophen. She was admitted for observation overnight, and thoracic surgery was consulted; a follow-up chest X-ray was recommended. Repeat imaging performed the next day showed a persistent pneumomediastinum that was slightly decreased as compared to the prior study (Figure 2).

The patient no longer endorsed chest pain or breathing difficulties remaining the next day. As a result, the patient and her mother were counselled on emergent symptoms that would warrant a return to the emergency department after discharge. The patient was also instructed to follow up with her primary care provider the following day.

## Discussion

Pathophysiology

Although the exact pathophysiology behind SPM is unknown, the development of a pneumomediastinum has been suggested to follow elevated pulmonary pressures that lead to alveolar rupture [5]. For this patient, it is suspected that the alveolar rupture was related to an abrupt change in pressure when swimming.

Free air may dissect the bronchovascular sheath and enter the mediastinum. This results in Hamman’s sign, a crunching sound with systole, and subcutaneous emphysema, symptoms commonly found in SPM patients. According to previous studies, subcutaneous emphysema is the most relevant sign that aids in diagnosis [6]. This clinical sign was also present in our patient. Non-specific pleuritic chest pain and dyspnea are also common in clinical presentation in SPM; however, these signs are not as specific [7].

In more complicated cases, the free air may penetrate the neck through communications of the mediastinum with the retropharyngeal and submandibular space [8]. SPM may be further complicated by decreasing cardiac output and causing cardiac tamponade, by compressing the larynx and causing stridor, or by mediastinitis due to a tear in the patient’s esophagus or alveolar rupture. Mediastinitis is a serious condition that carries high mortality if the patient is not treated properly or the condition is recognized too late [9]. Therefore, although most case reports solely observe SPM and provide supportive care, some recommend the administration of prophylactic antibiotics.

Treatment options

Not many protocols are available for the treatment of SPM. The treatment regimen is up to the discretion of the physician, with conservative therapy generally being utilized for SPM patients; however, empiric antibiotics are occasionally also included in management according to some case reports [9].

According to a meta-analysis, the survival rate of SPM is 92.5%, with no recurrence or complications; in 25.8% of patients, the patients required transfer to the intensive care unit [6]. This demonstrates that although supportive therapy is sufficient in most cases, it is crucial to observe the patient, in case the patient’s status declines. Clinically stable patients are typically under observation and should receive supplemental oxygen if indicated - ambulatory treatment may be appropriate in some patients who do not require supplemental oxygen providing close follow-up can be done. Another topic of debate is whether patients should receive prophylactic antibiotics as a precaution for the development of mediastinitis [9].

For most cases, conservative management with bed rest, oxygen inhalation - if needed, analgesics, and supportive care proves adequate. It is suggested to limit the use of empiric antibiotics to preventing infections when the patient presents with leukocytosis and fever [10].

If this patient’s alveolar rupture had been a result of swallowing water, prophylactic antibiotics could have been included in the treatment regimen due to the plethora of bacteria in a public pool to which the patient would have been exposed. Follow-up chest X-rays are debatable since most patients recover without complications. However, a patient with worsening or persistent symptoms should have follow-up chest X-rays and may require further radiologic studies.

## Conclusions

Early diagnosis of SPM is paramount in its management. Not all patients need hospitalization as very stable patients can be discharged with close follow-up. Others may need hospitalization for observation and possible further studies. To limit radiation in children, repeat X-rays may not always be needed but should be done if necessary. Most recommendations advise that empiric antibiotics should only be added in the case of leukocytosis, fever, or significant risk of infection. There is a lack of evidence to support the routine use of empiric antibiotics in SPM. Complications from SPM are rare to occur, rendering prophylactic antibiotics unnecessary in the majority of cases. Although no harm has been reported in any previous cases from this addition in the treatment course, there is simply no significant benefit. Given the findings, adopting the recommendation of only adding empiric antibiotics to conservative management if leukocytosis and fever are present is ideal until there is more concrete evidence supporting a certain treatment course.

Our patient had partial spontaneous resolution of SPM with supportive care, as evidenced in the second chest X-ray performed. Had our patient presented with leukocytosis and/or fever, the addition of prophylactic antibiotics would have been considered. Observation and conservative management of SPM without prophylactic antibiotics are just as safe and effective as with the inclusion of antibiotics in non-complicated cases.

## References

[1] Wong KS, Wu HM, Lai SH, Chiu CY (2013). Spontaneous pneumomediastinum: analysis of 87 pediatric patients. Pediatr Emerg Care.

[2] Ojha S, Gaskin J (2018). Spontaneous pneumomediastinum. BMJ Case Rep.

[3] Kouritas VK, Papagiannopoulos K, Lazaridis G (2015). Pneumomediastinum. J Thorac Dis.

[4] Takada K, Matsumoto S, Hiramatsu T (2008). Management of spontaneous pneumomediastinum based on clinical experience of 25 cases. Respir Med.

[5] Macia I, Moya J, Ramos R (2007). Spontaneous pneumomediastinum: 41 cases. Eur J Cardiothorac Surg.

[6] Gasser CR, Pellaton R, Rochat CP (2017). Pediatric spontaneous pneumomediastinum: narrative literature review. Pediatr Emerg Care.

[7] Newcomb AE, Clarke CP (2005). Spontaneous pneumomediastinum: a benign curiosity or a significant problem?. Chest.

[8] Zylak CM, Standen JR, Barnes GR, Zylak CJ (2000). Pneumomediastinum revisited. Radiographics.

[9] Gerazounis M, Athanassiadi K, Kalantzi N, Moustardas M (2003). Spontaneous pneumomediastinum: a rare benign entity. J Thorac Cardiovasc Surg.

[10] Kim KS, Jeon HW, Moon Y, Kim YD, Ahn MI, Park JK, Jo KH (2015). Clinical experience of spontaneous pneumomediastinum: diagnosis and treatment. J Thorac Dis.

